# Temporal scatterplots

**DOI:** 10.1007/s41095-020-0197-1

**Published:** 2020-11-07

**Authors:** Or Patashnik, Min Lu, Amit H. Bermano, Daniel Cohen-Or

**Affiliations:** 1 Tel-Aviv University, Tel-Aviv, Israel; 2 Shenzhen University, Shenzhen, China

**Keywords:** scatterplot, temporal data, visual clutter, principle component analysis (PCA)

## Abstract

Visualizing high-dimensional data on a 2D canvas is generally challenging. It becomes significantly more difficult when multiple time-steps are to be presented, as the visual clutter quickly increases. Moreover, the challenge to perceive the significant temporal evolution is even greater. In this paper, we present a method to plot temporal high-dimensional data in a static scatterplot; it uses the established PCA technique to project data from multiple time-steps. The key idea is to extend each individual displacement prior to applying PCA, so as to skew the projection process, and to set a projection plane that balances the directions of temporal change and spatial variance. We present numerous examples and various visual cues to highlight the data trajectories, and demonstrate the effectiveness of the method for visualizing temporal data.

### 1 Introduction

A central problem in data visualization is to plot multivariate data into a single map that conveys valuable information about the data. Plotting high-dimensional data on a 2D canvas is challenging, and a plethora of techniques have been developed to alleviate the innate clutter and reveal patterns in the data. Real-world data is often not just multivariate, but also varies over time, and the challenge in effectively presenting the time-series of the data is even greater. Many of the previous attempts to present temporal-multivariate data display a series of snapshots, presented side-by-side or as an animation, with special means used to allow the perception of data relations between and along the series [1–3].

In this work, we present a technique to visualize temporal-multivariate data in a single static plot. The key idea is to find a projection plane onto which the high-dimensional data may be projected, so as to best present the data trajectories. After using this plane to embed the data in 2D, a subset of trajectories is selected and visualized with enhanced strokes to visually convey the overall temporal progression. The guiding assumption here is that time is not just another dimension of the data, but a unique dimension along which other multivariate data is coherent, and visually perceiving the progression of the data over time is the main need.

In our work, we use principle component analysis (PCA) as the driving projection mechanism. The advantage of PCA is that it is linear, and thus facilitates intuitive interpretation of the data. Using PCA to project the data defines a 2D coordinate system, and a common projection plane, over which the data visualization plot is displayed. The key question is how to best define this projection plane.

As already mentioned, our premise is that in visualizing temporal data, the main objective is to clearly visualize the temporal changes, or *displacements*. However, there is a trade-off between the objectives of seeing the data points clearly, and perceiving the displacements well. The method that we present allows balancing of these two objectives, and defines a projection plane which explicitly considers both the displacements and the data distribution in the other dimensions.

Our approach is to define a projection plane by applying PCA on a manipulated version of the data. We create an intermediate representation by inflating

1 Tel-Aviv University, Tel-Aviv, Israel. E-mail: O. Patashnik, orpatashnik@gmail.com; A. H. Bermano, amit.bermano@gmail.com; D. Cohen-Or, cohenor@gmail.com.
2 Shenzhen University, Shenzhen, China. E-mail: lumin.vis@gmail.com (✉).
Manuscript received: 2020-07-22; accepted: 2020-09-12

each displacement, which accentuates the temporal changes, and then feed it to the PCA process. The original high-dimensional data is then projected onto this computed plane. Figure 1 demonstrates the effect of carefully setting the projection plane. The figure shows three embeddings of the same data, where in Fig. 1(a) the projection plane is defined by applying PCA over the data from all time-steps, in Fig. 1(b) the same data is embedded using t-SNE, and in Fig. 1(c) our method is employed. The data consists of time-varying 3D points, and is clustered into five different groups, each encoded with a different color. Two of these clusters have significant temporal change in the same direction. We show the points in the first time-step, and visualize trajectories for points which have significant displacement by drawing strokes from their positions in the first time-step to their positions in the second time-step. As can be seen, in Fig. 1(a) the temporal changes are not apparent, while in Fig. 1(b), although the temporal changes are clearly observed, their directions are opposite, which is misleading. Using our method in Fig. 1(c), parallelism is preserved and data presentation is balanced between spatial variance and temporal progression.

When there are several time-steps, the time series of a single data element forms a trajectory. To visualize the multitude of trajectories without creating visual clutter, we select a subset of the trajectories that well represent significant and interesting data progression, and display them with colored strokes (see Fig. 1). We evaluate our temporal scatterplots by showing their effectiveness for various datasets, and by comparing our visualization to established techniques. We analyze and discuss various design choices and demonstrate the usefulness of our method in conveying data progression.

### 2 Related work

In this section, we review related topics in both high-dimensional data visualization and temporal data visualisation.

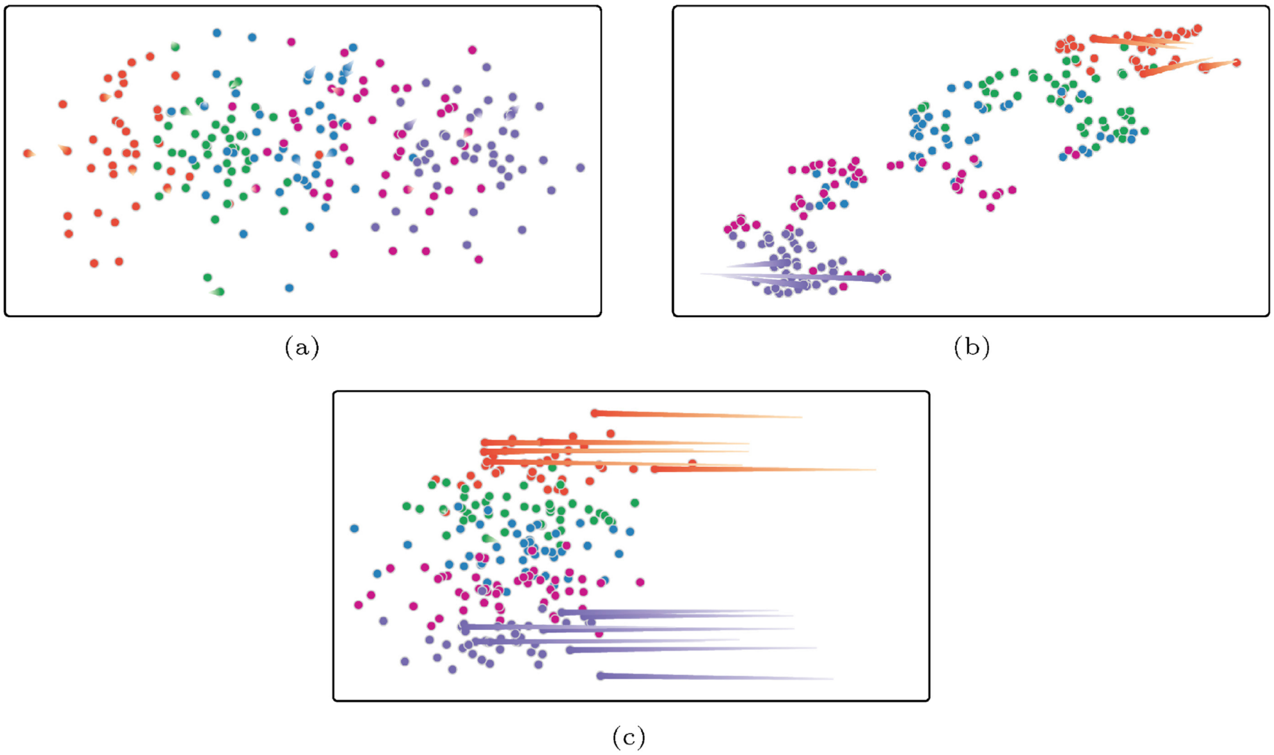

**Fig. 1** 3D temporal synthetic data, in two time steps, projected into 2D using different approaches. In the first time-step, the data is composed of five groups of normally distributed elements, whose means are co-linear. The colors indicate the different groups. For the second time-step, half of the points of two randomly chosen groups were significantly translated in a similar direction, while the remaining points were only slightly translated. Points in each figure correspond to the points in the first time-step. Strokes connect a point in the first time-step to the corresponding point in the second time-step. Strokes are drawn for a sample of the points which were traslated significantly. (a) A scatterplot visualization using PCA projection of the whole data. The same data embedded using (b) t-SNE, and (c) a projection plane computed by our method.

#### 2.1 High-dimensional visualization

Devising visual representations of high-dimensional data has always been one of the biggest challenges in the data visualization field [4, 5]. Many approaches to embeding high-dimensional data in a 2D or 3D space have been developed in recent decades. Some of the most common represent data elements as polylines across parallel dimension axes [6] or star plots [7]. Pixel-based visualization techniques separate the display by each dimension, and encode values using individual pixels [8]. Iconic figures (i.e., glyphs), such as Chernoff faces [9], can be used to depict high-dimensional data in a vivid manner. This work continues the topic of point-based high-dimensional visualization.

Scatterplots are the most popular point-based technique for visualizing high-dimensional data; data elements are represented as dots in 2D or 3D space. Stemming from the classic dot visualization, the visual design of scatterplots has been explored from multiple directions. Wang et al. [10] study coloring strategy for scatterplots to provide better perception of class separation. Mayorga and Gleicher [11] present Splatterplot, where dense point clouds are grouped into contours to visually emphasize classes. Lu et al. [12] attach wing-like mini-strokes to points in scatterplots to enhance multi-class perception. Chan et al. [13] draw small lines from points to depict local partial derivatives (i.e., sensitivity) linking one variable to another. These works are general visualization techniques to improve visual perception of scatterplots. Few of them take the temporal dimension as a specific design consideration. The core of our work is to find an overall projection of the temporal and high-dimensional data, which is not addressed by the aforementioned scatterplot enhancement techniques.

Scatterplots have been extended to handle high dimensions in many ways. A SPLOM (scatterplot matrix) organizes bivariate scatterplots into a matrix to support simultaneous observation of multiple bivariate relationships [14]. A family of SPLOM designs and interactions has been presented, such as matrix navigation [15], taking nominal attributes into account [16]. As the number of dimensions increases, the space of bivariate combinations quickly becomes too large for human comprehension. A SPLOM is often assisted with navigation interactions [15], or smart queries of the subspace [17]. Instead of showing every possible combination of two dimensions, scatterplots are more often used in combination with dimensionality reduction methods, such as PCA [18], t-SNE [19], MDS [20], UMAP [21], etc. For further details, we refer the reader to Notato and Aupetit [22], who surveyed various multidimensional projections, discussing their distortions and layout enrichment. Our work extends the conventional PCA with the temporal dimension and correspondingly presents a novel scatterplot method to visualize temporal high dimensional data.

#### 2.2 Temporal data visualization

When visualizing temporal data, there are two major ways to represent time [23]: one is to show the data frame by frame in an animated diagram, and the other is to represent data over all time steps in a static design. In accordance with human natural experience of time, animation easily catches researchers' attention and has been applied in visualizing temporal data many times in the past [24–26]. However, when going beyond just presenting the changes to assisting in high-level analysis tasks (e.g., tracking temporal trends, comparing temporal changes in multiple objects, etc.), animation encounters many challenges [27], especially keeping consistency over frames, e.g., keeping objects in the same group moving consistently [28], or applying smooth transitions between frames [29]. Without careful design, animation can be used poorly, because people have trouble tracking more than four moving objects at a time [30]. Unlike a dynamic solution for temporal data, our work seeks a static representation that squeezes dynamic features into a static visualization, for efficient observation of temporal trends and outliers.

Static visual design of temporal data has also received attention over the years. For example, Small Multiples [3] is a matrix of images depicting change over time, where each image presents a timeslice. Similarly, Rauber et al. [2] adapt t-SNE to maintain spatial coherence over plots for each time step. Juxtaposing images side by side supports easy comparison of temporal data [1], but it comes at the cost of mental effort to summarize temporal changes across multiple time steps.

Some work focuses on finding a common plot into which data from all time steps can be projected, while explicitly revealing the temporal features. For example, Crnovrsanin et al. [31] transform human indoor movements into a 2D space whose first dimension is spatial distance from a fixed point, and the other dimension is time. Jäckle et al. [32] present a visualization technique that computes temporal one-dimensional MDS plots for multivariate data which evolve over time. Later, Wulms et al. [33] introduce visual summaries using 1D orderings for entities moving in 2D based on several dimensionality-reduction techniques, such as PCA, Sammon mapping, and t-SNE. Unlike these works, which reduce high-dimensional data to a 1D representation and allocate the second dimension to time, our work steers the 2D PCA projection by temporal displacement, bringing the benefit of best balancing data variance and temporal change variance.

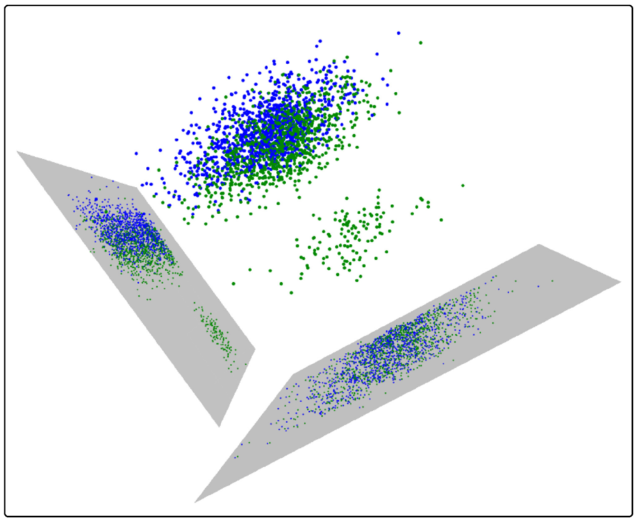

**Fig. 2** Embedding example of points in $\mathbb{R}^3$. The blue points are of the first time-step, and the green ones are of the second one. Two possible projection planes are illustrated. The right plane is selected by applying PCA over the whole data, and best illustrates the spatial arrangement of all the data. The left plane, however, is selected using our method, and focuses the visualization on the temporal evolution.

### 3 Method

We aim at visualizing high-dimensional data such that the temporal aspect of it is well perceived. To do that, the temporal aspect, or in other words—the motion, must be visible and consistent. The latter means that similar changes should also be presented similarly. Figure 1(b) highlights the importance of consistent visualization. The data points from the orange and purple groups appear to be moving in different directions, even though they are actually aligned. This occurs in methods that preserve relative distances (such as t-SNE [19] or UMAP [21]), and not directions. For this reason, we have chosen to base our method on the PCA technique. The method consists of two steps. In the first one we select the projection plane, and in the second we select the subset of trajectories to be rendered with an enhanced visualization.

#### 3.1 2D embedding

Figure 2 illustrates the difference between a naive PCA projection, where to the temporal evolution (transition from blue to green data points) is obscured, to the proposed one, in which this evolution is highlighted. The process of of defining the plane onto which the data is projected consists of three steps:

- Intermediate data generation. The given data is first manipulated and transformed to an intermediate data representation, where the temporal displacements are amplified.
- Projection plane definition. A PCA is applied to the intermediate representation to define the projection plane.
- Dimension reduction. The original data is projected onto the selected plane.

More formally, given $n$ trajectories of $D$-dimensional points, where each trajectory consists of $\tau$ time steps, we seek a 2-dimensional plane in $\mathbb{R}^D$, onto which the given points are to be projected, such that the prominent directions of change are expressed well along with the original spatial arrangement. We denote $p_i^t$ as the $t$-th time step of the $i$-th trajectory.

##### *3.1.1 Intermediate data generation*

In order to skew the PCA projection to the main directions of change, we propose to apply scaling along the trajectory, such that the displacements are amplified, while the variance in the spatial axes is insignificantly modified. This makes the variance along the temporal axis larger, and thus, the temporal changes more significant.

To do this, we first obtain the displacements along the temporal axis: For any $1 \leqslant i \leqslant n$ and $2 \leqslant t \leqslant \tau$ define the *displacement vector* $\delta_i^t$ such that:

$$\delta_i^t = p_i^t - p_i^{t-1}$$

Then, we scale each displacement vector by a *scale*

*factor* $\alpha$, and reconstruct the new points of each trajectory $T_i = (q_i^1 \cdots q_i^\tau)$ to reflect the introduced changes. Figure 3 depicts a fourfold trajectory undergoing this process, which can be done according to the following:

$$q_i^t = \begin{cases} p_i^t, & \text{if } t = 1 \\ q_i^{t-1} + \alpha \cdot \delta_i^t, & \text{otherwise} \end{cases} \quad (1)$$

We summarize some of our notations into a table:

| | |
|---|---|
| $n$ | Number of trajectories |
| $T_i$ | The $i$-th trajectory |
| $\tau$ | Number of time-steps |
| $p_i^t$ | The point of the $t$-th time-step in $T_i$ |
| $q_i^t$ | The point that $p_i^t$ was translated to |
| $\alpha$ | The scale factor |
| $length(T_i)$ | Sum of euclidean distances between consecutive points of a trajectory |

#### *3.1.2 Scale factor*

In the process of PCA, the principal components are the directions with the largest variance. Thus, scaling along the variance of the temporal changes highlights exactly these desired directions. This emphasis is controlled through the scale factor, $\alpha$. A larger $\alpha$ induces larger displacements and thus a direction that is more aligned with the temporal evolution. A smaller $\alpha$ would consequently give more attention to the spatial domain. In other words, by changing the value of $\alpha$ we control the ratio between the variance along the spatial axes, and that along the direction of temporal change. In Fig. 4, we show a series of results of our method, with increasing values of $\alpha$. As can be seen, with larger $\alpha$ values, the chosen projection direction is better aligned with the temporal evolution (and thus the motion trails grow), but is less aligned with the direction of spatial variance (and thus the data points cram together).

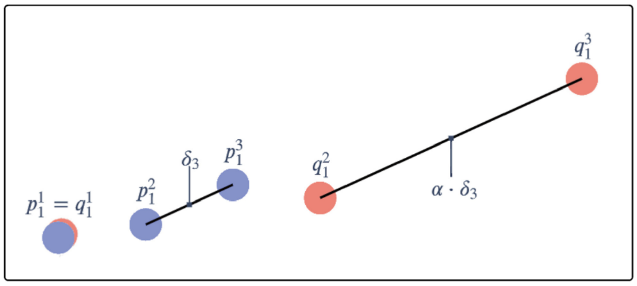

**Fig. 3** Displacement scaling. A trajectory of four time steps ($(p_i^t)_{t=1}^\tau$, purple) is transformed into our intermediate data representation ($(q_i^t)_{t=1}^\tau$, red). The first time step is kept in place. The rest are moved according to their amplified displacement. For example, the displacement between $p_i^2$ and $p_i^3$ ($\delta_3$) is depicted. After scaling, it remains in the same direction, but is $\alpha$ times larger.

Setting the right value of $\alpha$ depends on the data and context and expresses the trade-off between the time variance and spatial variance. Higher values of $\alpha$ should be chosen when highlight of the temporal change is desired. For example, in Figs. 11(b) and 10(b) the spatial axes have significant meaning—they provide the viewer with an orientation and geographical localization—which help in drawing more insights from the data. Hence, we chose a low $\alpha$, such that both the temporal changes and the geographic location are discernible. To facilitate choosing the right value for $\alpha$, we propose practical bounds for it. The lower bound is of course, 0, which eliminates temporal evolution altogether, and is equivalent to defining the embedding according to just the first time-step of the data. The value of 1 has more of a neutral meaning, giving no special attention to the temporal direction over the others, and is equivalent to applying PCA over the whole data. This value should be used when the temporal changes have no special interest.

For the upper bound, we propose a value such that increasing $\alpha$ over it has negligible effect on the result. We note that for very large $\alpha$ values, the displacements are large enough to undoubtedly become dominant during the PCA decomposition. Through this insight we define $\alpha_{\max}$, the highest value for $\alpha$ that would probably be required. The effect of increasing $\alpha$ above the $\alpha_{\max}$ is shown in Fig. 4. In this figure, synthetic data is projected into 2D using our method with different values of $\alpha$. As can be seen, setting $\alpha = 2\alpha_{\max}$ has almost no effect on the result, compared to $\alpha = \alpha_{\max}$.

We set the $\alpha_{\max}$ value to be one for which the spatial magnitude and the temporal change magnitude are even. To be more specific, for each pair of points that belong to the same time-step, we calculate their distance. Then, we set $\sigma$ as the standard deviation of those distances. Looking at a given trajectory $T_i$, we define its *length* as the sum of the euclidean distances between its consecutive points, and denote the euclidean distance by $\|\cdot\|$. We aim at setting $\alpha_{\max}$ the length of a trajectory after the amplification of the displacements is $\sigma$ on average.

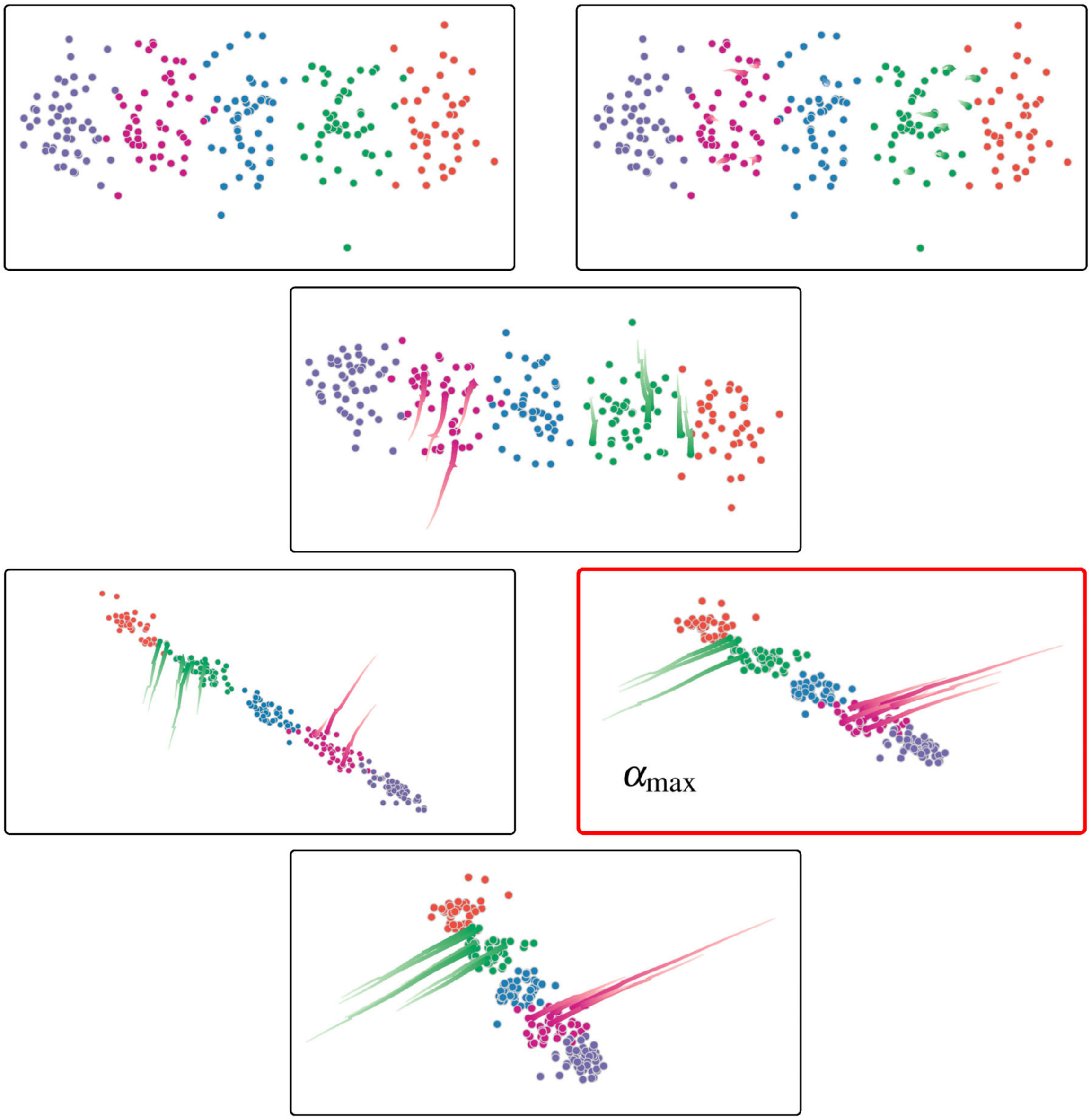

**Fig. 4** Demonstration of the scale factor $\alpha$ and its effect. From left to right and from top to bottom the $\alpha$ values are $0, 1, 4, 7, 20, 40$. The $\alpha_{\max}$ value computed for this data is $\alpha_{\max} = 20$, presented in the red frame. For $\alpha = 0$, the projection plane is equivalent to the one found by considering the first time-step alone, and hence the motion is hardly discernible. For $\alpha = 1$, the projection plane is equivalent to the one found by considering all the data together, giving the temporal axes no special attention. In general, the higher the $\alpha$, the more aligned is the projection plane to the temporal motion. Using higher values than $\alpha_{\max}$ yields very little change, as can be seen in the last example.

In other words, we wish for the following:

$$\text{mean}\left(\sum_{t=1}^{\tau-1} \|q_i^{t+1} - q_i^t\|\right) = \sigma$$

By the definition of $q_i^t$ we get that $\|q_i^{t+1} - q_i^t\| = \alpha_{\max} \|p_i^{t+1} - p_i^t\|$ and therefore:

$$\sum_{t=1}^{\tau-1} \|q_i^{t+1} - q_i^t\| = \alpha \sum_{t=1}^{\tau-1} \|p_i^{t+1} - p_i^t\| = \alpha \cdot length(T_i)$$

To obtain $\alpha_{\max}$ we divide $\sigma$ by the mean length of the trajectories before the amplifications, i.e., $\alpha_{\max}$ is set as

$$\alpha_{\max} = \frac{\sigma}{\frac{1}{n}\sum_{i=1}^{n} length(T_i)}$$

#### 3.1.3 Discussion

The last step is rather intuitive. After obtaining the modified trajectories $T_i = (q_i^1 \cdots q_i^\tau)$, we apply dimension reduction using PCA and find the plane onto which to project the points. Then, we project the original points of each trajectory, $(p_i^1 \cdots p_i^\tau)_{i=1}^n$, onto this plane. Note that even though the plane was found using the intermediate set $\left(q_i^1 \cdots q_i^\tau\right)_{i=1}^n$, the direction of the temporal changes of these intermediate points coincides with that of the original data. This means that we project onto a direction that is aligned better with the temporal behaviour, but still respects the spatial distribution.

Note that there are many other ways through which one could perform a 2D embedding for a given data set, such that the temporal aspect of it is emphasized. In the following, we list a few plausible alternatives, and discuss their differences in relation to the proposed approach. First, as already mentioned, one could imagine using a non-linear method, such as t-SNE [19] or UMAP [21]. While these methods aim at reflecting respective distributions between points they do not preserve directions in general, which means that directions of motions are distorted. Figures 1 and 7 convey this notion. In Fig. 1(b), a t-SNE visualization is used. Due to the inherent distortion, the motion of the orange group and the purple one seem opposite, even though they are of very similar directions in 3D. Our visualization (Fig. 1(c)) depicts exactly that. The same effect can be seen in Fig. 7(c), where the green group seems to be moving in different directions, when in fact they do not—as can be seen by our visualization in Fig. 7(d).

Even when discussing a PCA-based embedding, many different options can be considered for substituting our approach. Naively, one could use the first time-step, or the whole data together, to find the projection plane. These approaches are equivalent to using $\alpha = 0$ and $\alpha = 1$, respectively, and their implications on the visualization are discussed in Section 3.1.2. Another idea could be taking the first principle component of the spatial distribution (e.g., by considering only the first time-step), and the first one of the temporal motion (e.g., by considering only displacements). This, of course, is prone to failure due to several reasons: The two mentioned directions could be close to parallel, there could be two important temporal directions to be shown, the average of the two directions could be enough to depict the information, and many other similar examples and degeneracies. In addition, blending the two directions to achieve balancing between the temporal and spatial directions is unclear, since averaging, or rotating between the chosen directions is ambiguous.

Finally, a prominent alternative approach would be to facilitate Weighted-PCA (WPCA) [18]. In principle, WPCA was developed to address data samples with varying quality, by giving less informative samples less weight [34]. One could harness this approach by viewing the trajectory length as quality—the longer the length, the higher the weight of the points in the corresponding trajectory. Balancing the spatial and temporal directions is also straightforward in this approach, since it can be directly controlled through the magnitude of the weights. This approach, however, has a fundamental flaw, which our proposed method does not—the weighing process skews the spatial directions of the data, and does not just accentuates the temporal one. This phenomenon is depicted in Fig. 5. In this figure, a synthetic data-set is depicted, for which there is a clear primary principle direction (along which the red points are scattered). Our approach (Fig. 5(c)) with large $\alpha$ values generates a rendering that emphasizes the temporal evolution direction and the primary spatial component, as one would expect. Using WPCA, however, gives more weight to all the points in green, so instead of amplifying only the temporal direction, it also assigns additional importance to the secondary spatial principle direction, along which the green points are scattered. Hence, the visualization shown in Fig. 5(b) depicts the temporal direction, like expected, but it also undesirably depicts the less significant spatial direction.

#### 3.2 Visualization of trajectories

To visualize the progression of the temporal data, we display a selected number of trajectories with enhanced graphical strokes—a tail-like design which traces the temporal change by gradient color. To avoid visual clutter that may be caused by visualizing all or a large number of trajectories, we carefully choose a subset of trajectories to display.

The selected subset of trajectories is meant to express the variety of progression directions, focusing on the more outstanding ones, and at the same time avoiding visual clutter. In particular, we prioritize long trajectories since these typically stand out, and contribute to the overall impression of the data progression. To prevent clutter, we avoid selecting nearby trajectories that represent similar trajectories.

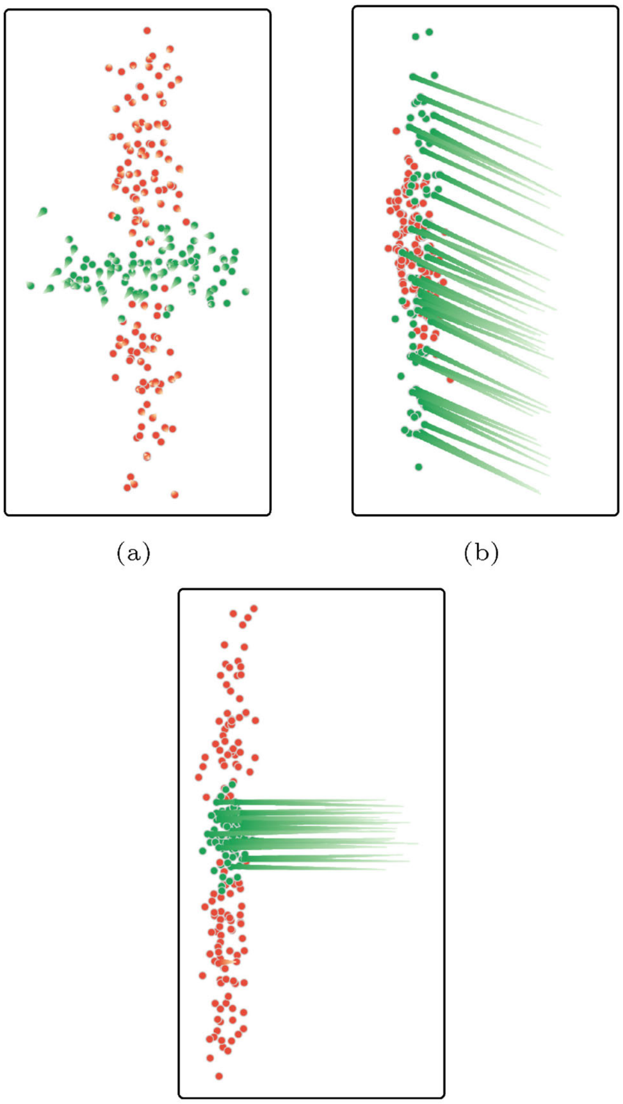

(a) (b)

(c)

**Fig. 5** Toy example depicting the usage of weighted PCA (see Section 3.1.3). The data is distributed along two spatial lines, where one (red points) is more dominant than the other (green). The temporal motion is mostly orthogonal to both directions, and is significantly larger for the green points. (a) Visualization of the data using our method, with $\alpha = 1$, for clarity. (b) Visualization using a weighted PCA method, where the weight of a point is proportional to its trajectory length. As can be seen, the secondary spatial axis has been chosen for visualization, even though it depicts the spatial arrangement less, since the green points receive more weight. (c) Visualization using our method, with $\alpha = \alpha_{\max}$. Since only the motion has been amplified, and not the spatial aspect of the green points, the visualization is not biased, and hence is aligned with both the temporal direction and the primary principle direction of the data.

To select which trajectories to highlight, we define a probability, $p_i$, for each trajectory $T_i$. Longer trajectories should have increased probability to be selected, while other trajectories that are similar should decrease it. In our implementation we give more weight to the number of similar trajectories than their length. To count the number of similar trajectories to a given one, we define a distance measure, and threshold it.

In cases where the data consists of several classes, it is desirable to consider every trajectory relatively to the other ones in its class. This encourages displaying temporal changes of all the classes, even if these include similar trajectories across classes.

To calculate $p_i$ we look at the trajectory after it was reduced to be 2-dimensional. Recall that for a trajectory $T_i$ its initial point is denoted by $q_i^1$ and its last point is denoted by $q_i^\tau$. First, for any $i, j$ we define their similarity, $d(T_i, T_j)$ (see Fig. 6). For $0 \leqslant l \leqslant 1$ we denote by $r_i^l$ the point for which the length of the trajectory from $q_i^1$ to $r_i^l$ is $l \cdot length(T_i)$ where $length(T_i)$ is defined as in Section 3.1.2. We set $d(T_i, T_j)$ as

$$d(T_i, T_j) = \sum_{k=0}^{\tau} dist\left(r_i^{k/\tau}, r_j^{k/\tau}\right)$$

where $dist$ is the euclidean distance metric. We then define $s_i$, which is the number of trajectories that are similar to $T_i$ by counting the number of trajectories $T_j$ for which $d(T_i, T_j) < \Gamma$, where $\Gamma$ is a fixed predefined scalar. Then, we denote $\overline{s_i}$ and $\overline{length(T_i)}$ the normalized $s_i$ and $length(T_i)$ obtained by dividing in $\max_i\{s_i\}$ and $\max_i\{length(T_i)\}$ respectively. For the case where we want to have probabilities that are relative to the class, we perform another step. We multiply the probabilities of each class in some constant, such that the probabilities within this class are summed to the number of trajectories that we desire to display from this class. The number of

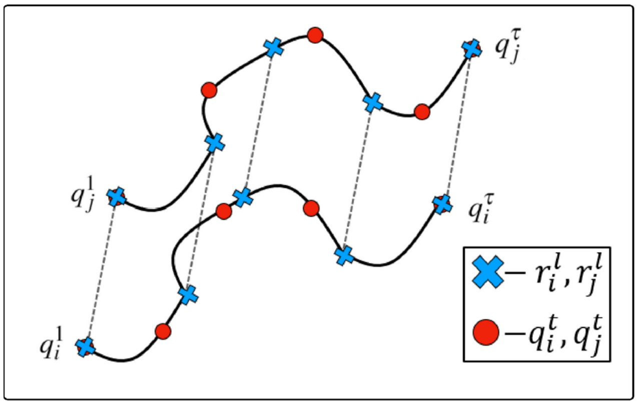

**Fig. 6** To determine whether trajectories $T_i$ and $T_j$ are similar, we uniformly re-sample the curve, and sum the distances between the data new sample points (blue crosses). These distances are depicted by the dashed lines.

trajectories that are desired to be displayed is equal for all the classes. Finally, for each trajectory $T_i$ we define its probability to be selected as follow:

$$p_i = \frac{\log\left(\overline{length(T_i)} + 1\right)}{2(\overline{s_i} + \varepsilon)}$$

where $\varepsilon$ is a small number that depends on the data and ensures that we do not divide by zero. We set $p = 1$ if the calculated value is greater then 1.

## 4 Results

To test our method, we applied it to various datasets, one synthetic, and two real world. In the following, we describe the datasets and show the results of applying our method to visualize them. We also consider alternative approaches.

### 4.1 Synthetic data

#### *4.1.1 Procedure*

In this example, we applied our method to a synthetic dataset, and compared it to alternative procedures of dimensional reduction to $\mathbb{R}^2$: (i) apply PCA to the whole data and project the data onto the obtained planen; (ii) apply t-SNE; (iii) apply UMAP.

We synthesized data as follows. First, we created the first time step by sampling five 3-dimensional Gaussians, with centers one next to the other. For each consecutive time step, we first randomly chose a subset from the dataset. For this subset, we chose an arbitrary direction, and moved the data along this direction. For the rest of data, we added a small random disturbance only. Some results using this data are displayed in Figs. 7 and 9, where each row in Fig. 7 uses a different dataset. The presented results contain six time steps, with only a small sample of the dataset points being shown. The different colors refer to different Gaussians.

#### *4.1.2 Comparison to other methods*

In each column in Fig. 7 the data were projected using a different method. In the first column (Figs. 7(a) and 7(e)), we applied PCA to the data from all time steps. As can be seen, the magnitude of the temporal changes captured by the PCA is smaller than their actual magnitude. In the second and third columns (Figs. 7(b), 7(f), and 7(c), 7(g)), we projected the data using t-SNE and UMAP, respectively. These methods lost the spatial structure of the data, and although temporal changes can be seen, their directions do not reflect the direction of temporal changes in the data. The last column shows the results of our method. As can be seen, our method keeps both the spatial structure and highlights the magnitude of the temporal changes.

#### *4.1.3 Designs*

Depending on the data, one may want to highlight different properties of the temporal progression. For example, if we seek the temporal behavior of different entities in the data at a certain time, it is important that the time, or velocity of the trajectories, can be inferred from the stroke. In some cases the first point of the trajectory is the reference point, while in other cases it is the last one. Thus, we propose different designs that can be used in different cases and show their results on our synthetic data. Figure 9 proposes some designs.

In Fig. 9(a) the color and width of the trajectory are related to its progression along the trajectory. In this design we highlight the magnitude of the temporal change at the exact time-step it occurs in. In Fig. 9(b) we use constant colors for each time segment, i.e., for trajectory $T_i$, the color of the segment connecting $p_i^t$ to $p_i^{t+1}$ is the same throughout the segment. This enables distinguishing of velocities, or time steps for which the temporal change is larger or smaller. In addition, using this coloring scheme, we can compare the same time-step for different trajectories. The width of each segment varies along the segment to further highlight each time segment. In Fig. 9(c), the width is relative to progress along the trajectory, but the color performs a full gradient transition at every segment. This visualization highlights the association of points with their trajectories even further.

### 4.2 Hans Rosling's statistical data

In this example, we regenerate Hans Rosling's famous visualization of country statistical data, but in a static form. For 144 countries, in the years 1960, 1970, 1980, 1990, 2000, 2010 the following data were collected: population size, life expectancy, and GDP. Therefore, we have temporal data with six time steps in 3 dimensions. The general trend in these three dimensions is increasing. This trend is more evident in the life expectancy and GDP measures. Although the size of the population increases, it is less strong than the increases in the other two measures. The

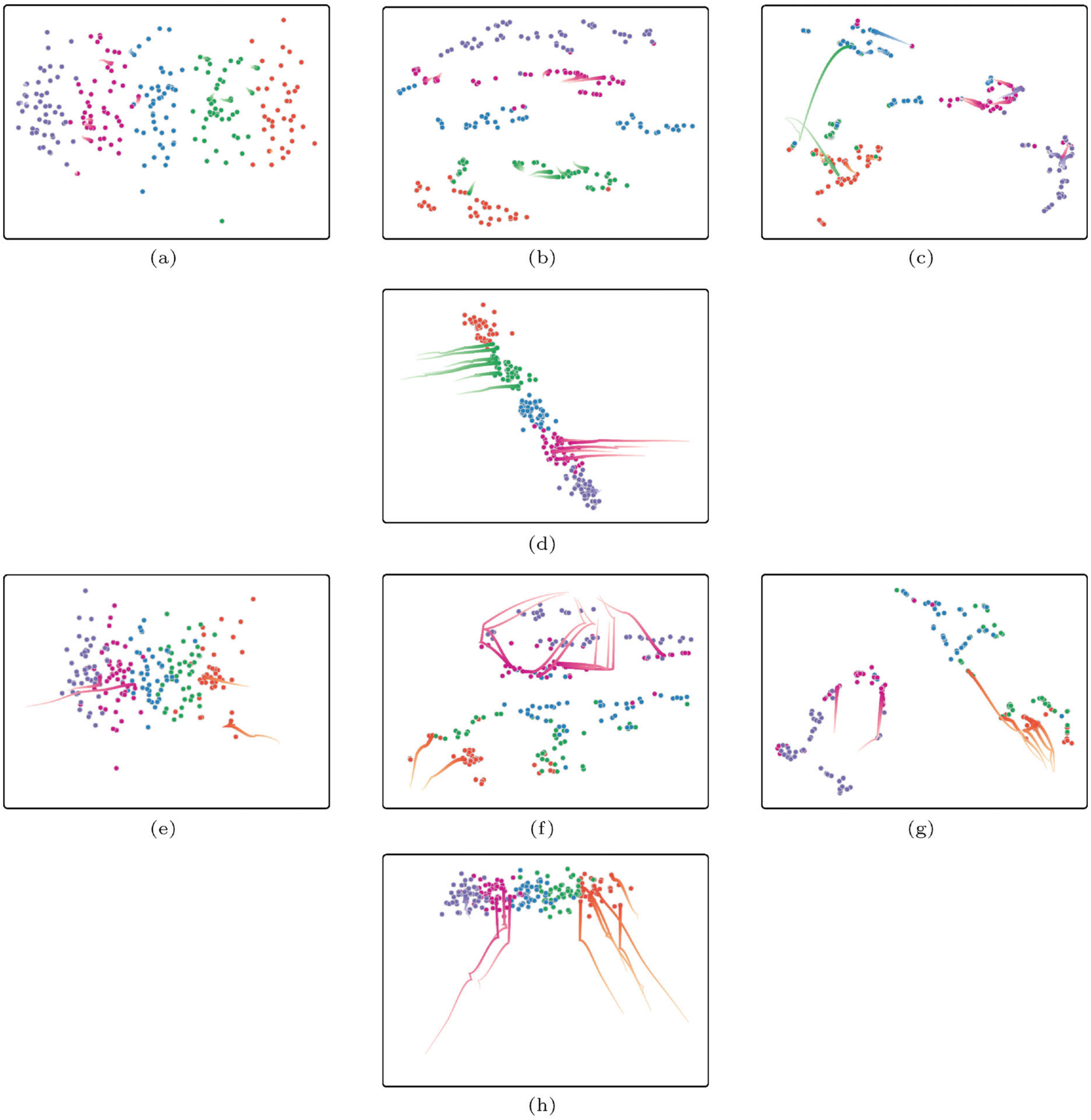

**Fig. 7** Synthetic datasets embedded in 2D using four different methods: (a, e) naive PCA, computed over all time steps together, (b, f) t-SNE, (c, g) UMAP, (d, h) our method. Note how temporal aspects are hardly visible using naïve PCA, and how the t-SNE and UMAP methods do not preserve directions, making parallel trails seem dissimilar.

data is plotted in Fig. 8, where the semantic meaning of the axes is defined heuristically.

In Fig. 8(a) the data is projected using PCA applied to the whole data, while in Fig. 8(b) the data is projected using our method. In Fig. 8(a), it is clear that there is temporal change, but it seems as if it has only one significant direction. Figure 8(b) expresses well the fact that there are two significant directions for the temporal change.

In Fig. 8, each color refers to a different continent (Africa, Asia, Europe, and North America). The vertical axis corresponds approximately to the GDP, and the horizontal axis corresponds approximately the life expectancy. It is clear that the countries of Africa, Europe, and North America have similar measurements and thus the countries in these continents are grouped and the direction of their temporal change is similar. The countries of Africa have significant growth in the life expectancy, while almost no growth in GDP. The countries of Europe

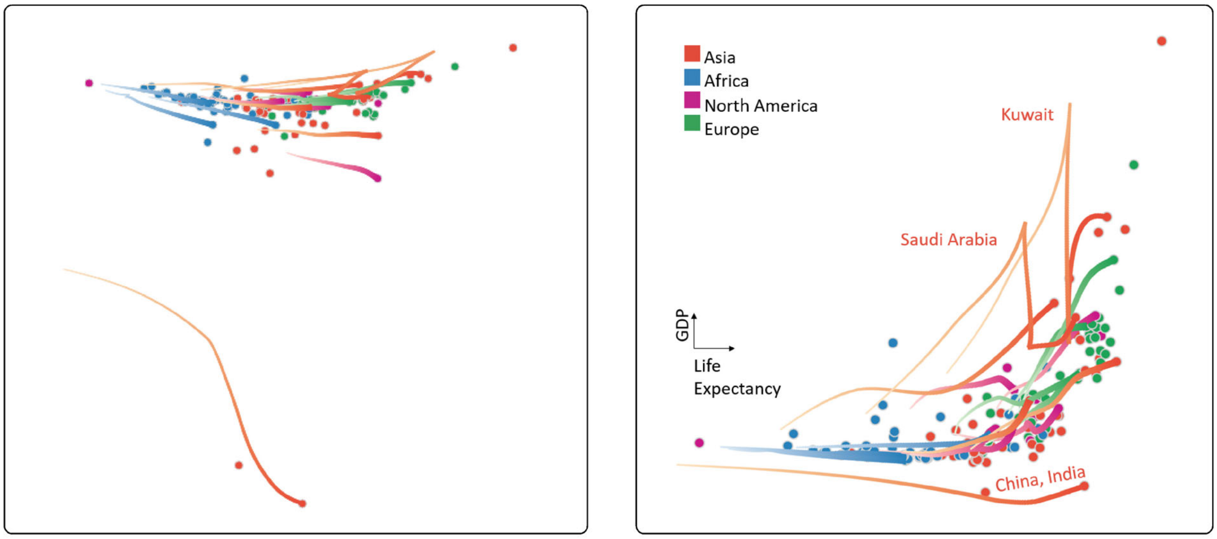

(a) (b)

**Fig. 8** Hans Rosling's statistics. Points represent three statistics of countries (population size, life expectancy, and GDP) over six selected years: (a) projected by PCA over the whole data, (b) projected using our method. Marked points represent the last time step, the stroke getting wider as time passes. Choosing strokes with respect to the class was used to get insights on each class separately.

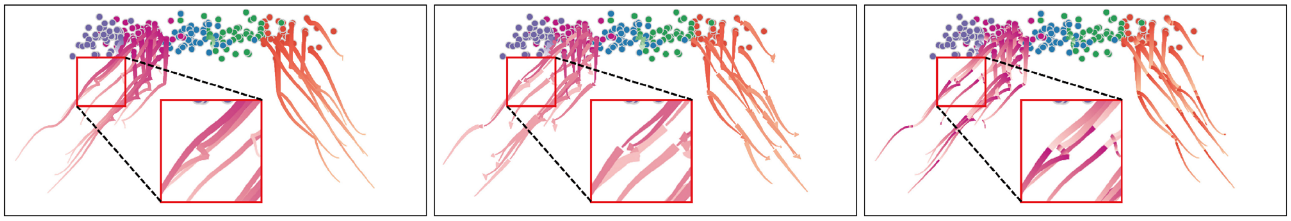

(a) (b) (c)

**Fig. 9** Synthetic data projected into 2D using our method. In each case the stroke's design is different: (a) smooth color and width transitions, (b) piece-wise constant coloring with a repeating thickness pattern, (c) repeating coloring pattern with a smooth width transition.

and North America have significant and persisting growth in GDP; it can be observed that most of those countries are developed countries. In the countries of Asia there is a variety and it is harder to identify common trends. There are two prominent groups in the countries of Asia. First, consider the two points at the bottom, which correspond to China and India. The populations of those countries are clearly the largest in the world and significantly larger than all other countries. Therefore, these countries are projected to a different region away from the other countries. The location of these points in the plot is related to the population axis in 3 dimensions, and therefore the approximated axes are not relevant for these points. Another prominent group that can be observed in the visualization is countries with a very high GDP, which then shrinks. Countries with this trend are Brunei, Kuwait, Qatar, Saudi Arabia, and United Arabs Emirates. Their peaks in GDP are related to the 1980s oil glut.

#### 4.3 COVID-19 data

In this example, we demonstrate the effectiveness of our method in providing insights into COVID-19 related data. We show data from China and the USA. For each country, we take data at province or state level, respectively.

For China, we considered January 22nd to 31st, 2020, and take as dimensions the geographic coordinates of each province, the number of confirmed cases, the ratio of people that entered the province from Hubei, and the ratio of people that entered the province from the rest of China. Due to lack of symmetry, we did not include Hubei in the plot. Thus, this data is 5-dimensional with 10 time-steps, and is depicted in Fig. 10. In Fig. 10(a) the projection is performed by applying PCA to the whole data. It can be seen that only the geographic location is expressed, and therefore the scatter plot resembles the map of China, with a point in the center of each province. In Fig. 10(b) the data is projected using our method. The PCA process highlights axes which are in essence the number of confirmed cases and the ratio of incoming population. We see that most prominent provinces in the figure are those which are close to Hubei. This confirms that the provinces with a large number of cases are those close to Hubei. In addition, by looking at the data, we may infer that Guangdong had an increasing amount of incoming population, also making this province prominent.

For the USA, we looked at data from March 8th to 13th, 2020. The axes taken were the geographic coordinates of each state, and the number of confirmed cases. Therefore, the data here is 3-dimensional with 6 time-steps. The data can be seen in Fig. 11. In Fig. 11(a) the data is projected by applying PCA to the whole data. As in Fig. 10(a), the geographic axes are the principal components

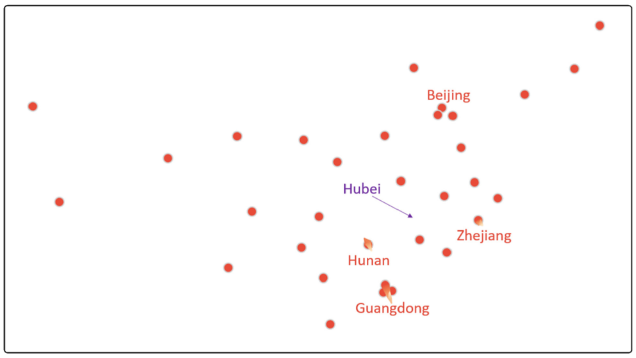

(a)

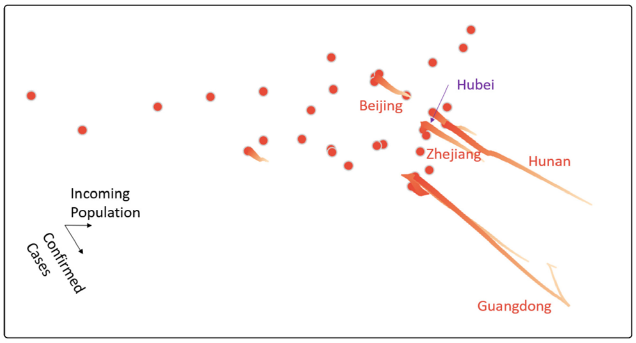

(b)

**Fig. 10** COVID-19 data in China. Each point is a province in China, with five dimensions (longitude, latitude, immigration ratio from the whole country, immigration ratio from Hubei (from 2020-01-22 to 2020-01-31): (a) applying PCA to the whole data, (b) using our method to project the points.

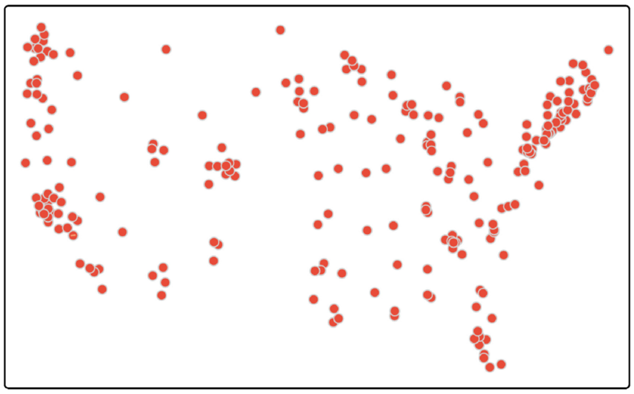

(a)

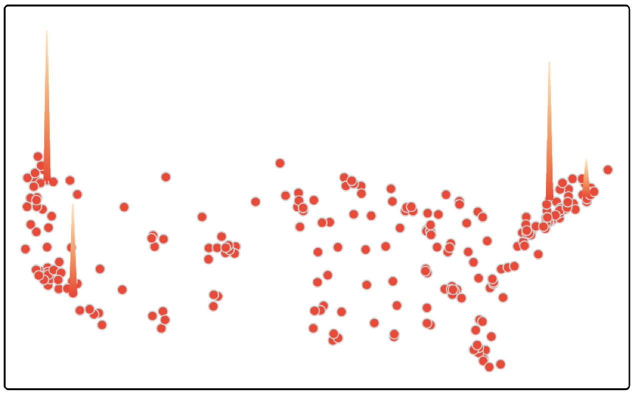

(b)

**Fig. 11** COVID-19 in the USA. Each datum represents a state, with 3 dimensions (longitude, latitude, and confirmed case) in the second week of March: (a) projected using applying PCA over the whole data; (b) projected using our method.

and no change in the number of confirmed cases can be observed. In Fig. 11(b) the data is projected using our method. In this figure it can be seen that the number of confirmed cases is increasing, while it is also possible to recognize the US map. Using insights into both geography and number of cases, it is possible to understand that the epidemic spread started from the East coast and West coast: more specifically, New York, Washington, and California.

### 5 Evaluation

Assessing the efficacy of different visualization methods for temporal data, e.g., animation, small multiples, and other non-animated variants, has been long studied and a body of experimental research has been carried out. A closely related study, which is important to our work, was carried out by Robertson et al. [35]. In their experiment, they thoroughly examined the performance of three different visual forms (animation, small multiples, and a static trail) in visualizing Rosling's dataset. They found that animation is more fun and exciting, while non-animated visualization, e.g., using small multiples, allows completes tasks such as trend comparison to be performed more efficiently than an animated design.

In this work, rather than reproducing Robertson et al.'s study, we focus on advocating the new PCA projection method adapted for temporal data. Therefore, a full experimental comparison of our temporal scatterplots with animations is outside of the scope of this work. Instead, we performed a small pilot study to understand better the advantages and disadvantages of our method.

We performed a qualitative user study with 10 participants from our local university. All of them were graduate students in computer science, with no or little knowledge of visualization. In the experiment, we introduced them to Rosling's examples in two forms, animation and our static scatterplot. We chose Rosling's dataset for the experiment because it is easy for participants to quickly understand the data. For each participant, we showed them both the animation and our static version in a random order. For a fair comparison, we played the animation of Rosling's scatterplot with voices muted. Then we interviewed the participants by asking a series of questions, such as *which visual form do you prefer?*, *what insights can you get from the visualizations?*, *what (dis)advantages do you see of each visualization?*, etc.

In the result, most participants (8/10) reported that animation is more interesting and more comprehensive than the static plot. One participant commented that "it is more fun to see animation!" Three participants complained that it took them a while to understand our scatterplots. Considering relative merits of both forms, several participants (6/10) said that more details can be found in the animation than in the plot, such as the changing trace of each country, etc. This finding is in line with Robertson et al.'s finding that animation can better support lower-level tasks. Most participants (9/10) agreed that our static temporal plot provides a better summary in a short time than the animation. One participant specifically said "it is very impressive to see the groups with similar trends at a first glance in the plot, which are pretty hard for me to identify in the animation". Our findings in this small user study are basically aligned with the conclusions drawn by Robertson et al. Animation can be associated with lower-level task performance relative to a static alternative, e.g., tracking one or two objects over time, comparing data between two sequential time steps, etc. Our scatterplots are better at providing an overall summary of the temporal data, especially showing objects with similar trends over time.

### 6 Conclusions

In this paper we presented a technique to visualize temporally progressing high-dimensional data on a single static chart. This type of data is commonly found in numerous real-life cases, where corresponding samples are measured over time. More generally, the temporal dimension can be replaced with any dimension that represents a continuum with dependent behavior. Being of ample relevance, many approaches have been proposed to visualize such data. All of these approaches complicate the viewing process, either by presenting the data over multiple charts, or by using animations, which take longer to process and require special media. Quite surprisingly, little attention has been given to depicting temporal evolution on a single chart, without explicitly having time as one of the plot axes.

The key insight of our method is in the embedding stage, where we magnify the temporal change by scaling displacements, thus affecting the process of selecting the plane of projection. Additionally, we offer the user natural, conveniently bounded, control over the balance between temporal progression and spatial variance. To visualize the progression after embedding, our method draws inspiration from the animation domain, where motion is expertly expressed. The adopted principles of drawing a single line to represent the motion of a group, or fading a trail as it grows farther from the point of interest, are well established in the animation field. The combination of these two stages offers an intuitive and simple to implement method. We have demonstrated the former by exhibiting how insights can be drawn intuitively from the visualization, without any training or explanation. Finally, we have explored several other plausible alternatives to our approach, and have shown how our method alleviates their disadvantages.

The proposed method, of course, poses some assumptions about the data. If the directions of evolution are aligned with the spatial variance, or if they are independent and varied, our approach has little advantage. In the latter case, many motion directions will actually be omitted, in order to avoid clutter, and hence will not be conveyed at all. In the future, we would like to examine different approaches to group similar trajectories. These could be through a group-size related thickness, or by visually merging similar trajectories into one trail. Another approach could involve selecting which trails to show; careful consideration of trail intersections could reduce clutter, and a perception-based understanding of outstanding or representative trails could convey the information better for the same number of displayed trajectories. Finally, our work relies on linear projection of the data. Extending the proposed notions to established non-linear embedding procedures (e.g., t-SNE) would be a non-trivial but noteworthy objective. No matter in what direction, we believe this research will open up many different ways to efficiently represent temporal progression in a static chart.

### Acknowledgements

This work was supported in part by the Israel Science Foundation (Grant No. 2366/16 and 2472/17).
